# Couples HIV counselling and couple relationships in India, Georgia and the Dominican Republic

**DOI:** 10.1186/s12889-017-4901-8

**Published:** 2017-11-25

**Authors:** Thierry Tiendrebeogo, Melanie Plazy, Shrinivas Darak, Marija Miric, Eddy Perez-Then, Maia Butsashvili, Patrice Tchendjou, François Dabis, Joanna Orne-Gliemann

**Affiliations:** 1 INSERM UMR 1219 – Bordeaux Population Health Research Center, Bordeaux, France; 2 Universite Bordeaux, Institut de Santé Publique, d’Epidémiologie et de Développement (ISPED), 146 rue Leo Saignat, 33 076 Bordeaux cedex, France; 3 Prayas Health Group, Pune, India; 4 Global Health and Biotechnology Research Center, O&M Medical School, Santo Domingo, Dominican Republic; 5 Health Research Union, Tbilisi, Georgia; 6 Laboratoire d’Epidémiologie et de Santé Publique, Centre Pasteur du Cameroun, Yaoundé, Cameroon; 7 Réseau International des Instituts Pasteurs, Paris, France

**Keywords:** HIV, Couples HIV counseling, Antenatal care, Male partner

## Abstract

**Background:**

Couples HIV counseling and testing is essential for combination HIV prevention, but its uptake remains very low. We aimed to evaluate factors associated with couples HIV counseling uptake in India, Georgia and the Dominican Republic, as part of the ANRS 12127 Prenahtest intervention trial.

**Methods:**

Pregnant women ≥15 years, attending their first antenatal care (ANC) session between March and September 2009, self-reporting a stable partner, and having received couple-oriented post-test HIV counseling (trial intervention) were included. Individuals and couple characteristics associated with the acceptability of couples HIV counseling were assessed using multivariable logistic regression for each study site.

**Results:**

Among 711 women included (232, 240 and 239 in the Dominican Republic, Georgia and India, respectively), the uptake of couples HIV counseling was 9.1% in the Dominican Republic, 13.8% in Georgia and 36.8% in India. The uptake of couples HIV counseling was associated with women having been accompanied by their partner to ANC, and never having used a condom with their partner in the Dominican Republic; with women having been accompanied by their partner to ANC in India; with women having a higher educational level than their partner and having ever discussed HIV with their partner in Georgia.

**Conclusion:**

Couple HIV counseling uptake was overall low. Strategies adapted to local socio-cultural contexts, aiming at improving women’s education level, or tackling gender norms to facilitate the presence of men in reproductive health services, should be considered.

**Trial registration:**

ClinicalTrials.gov Identifier: NCT01494961. Registered December 15, 2011. (Retrospectively registered).

## Background

Worldwide, an estimated 2.1 million people became newly infected with HIV infection in 2015 [1]. A very large part of these new infections occurred within heterosexual and serodiscordant stable couple relationships [2], as evidenced by the abundant literature from sub-Saharan Africa [3–6]. Several Asian countries have also reported high levels of intimate partner transmission of HIV [7], with women living with HIV being infected by their married partner who engaged in unsafe behaviors, prior or after marriage [8]. Preventing HIV transmission among serodiscordant couples thus remains a key target in the worldwide fight against HIV.

Significant new advances have taken place in the past years in the field of HIV prevention research, mainly in the field of biomedical interventions for serodiscordant couples. The HPTN052 trial showed that when an HIV-infected person is on antiretroviral (ARV) treatment (ART) with good adherence, his/her risk of HIV transmission to his/her uninfected partner is significantly decreased [9]. Within the PARTNER Pre-exposure Prophylaxis (PreP) study, when the HIV-negative partner used ARV drugs as prophylaxis, his/her risk of HIV acquisition from the HIV-infected partner was significantly reduced [10]. This evidence contributed to the revision of the World Health Organization guidelines in September 2015, which recommend initiation of ART to all HIV-infected people regardless of their immunological status, as well as ARVs as prophylaxis for serodiscordant couples [11].

Implementing biomedical interventions aiming at preventing HIV acquisition and transmission within couples requires a first critical step, i.e. HIV status awareness within couples. Couple members who get tested together and mutually disclose their HIV status are more likely to adopt HIV prevention behaviors, both in HIV concordant or discordant relationships [12–17]. Further, women who receive prenatal HIV counseling along with the partner are more likely to adhere to prevention of mother-to-child transmission (PMTCT) interventions when they test HIV-positive than those who are counseled individually [18].

Couples HIV counseling and testing has been encouraged by international agencies for many years [19], but efforts have mainly been restricted to the context of generalized HIV epidemics in sub-Saharan Africa [20, 21]. Extensive research has been conducted on couples HIV counseling in Rwanda and Zambia, including on promotion strategies [22, 23] and on the profile of couples accepting such services – in Zambia, having been previously tested for HIV and cohabiting were among the main factors associated with couples HIV counseling uptake [23]. In Zambia, couples HIV counseling was shown to be associated with sustained reductions in self-reported unprotected sex [24]. High rates of couples HIV counseling were recently reported at national level in Rwanda, preventing an estimated >70% of incident HIV infections [25]. In most countries however, the proportion of couples who test together in the context of pregnancy is less than 20% [26], and the provision of couple HIV counseling and testing services is low [27].

There is little data on the uptake of couples HIV counseling and testing in low to medium HIV prevalence countries and outside sub-Saharan Africa. And yet in such settings, low coverage of HIV testing clearly represents missed opportunities for preventing primary acquisition of HIV within couples. Further, the role of different types of couple relationships and gender norms, and of the local social and epidemiological contexts, on the acceptability of couple approaches to HIV prevention is poorly understood.

This paper aims to describe the uptake of and factors associated with couples HIV counseling in low HIV prevalence settings.

## Methods

### Study setting: The 12,127 Prenahtest trial

We conducted this analysis using the data collected in the ANRS 12127 Prenahtest study, a multicenter randomized controlled trial carried out between 2009 and 2011 in four urban health facilities located in Yaoundé (Cameroon), Santo Domingo (The Dominican Republic), Tbilisi (Georgia) and Pune (Maharashtra province, India). In 2014, HIV prevalence among adults aged 15–49 was estimated at 0.3% in Georgia, 0.4% in Maharashtra province, 0.8% in the Dominican Republic in 2014 and 4.8% in Cameroon [28].

Trial methods were described previously [29]. In summary, the trial was designed to evaluate the acceptability, feasibility and impact of an innovative prenatal counseling intervention offered to pregnant women, named Couple-Oriented post-test HIV Counseling (COC). The main study outcomes were the frequency of men’s HIV testing, couples HIV counseling and couples HIV testing, as well as sexual, reproductive, and HIV prevention behaviors. Pregnant women were recruited during antenatal care (ANC). The trial baseline criteria were acceptance of trial participation, being aged 15 years or older, attending their first ANC visit in the four study centers between March and September 2009, no previous HIV test during their current pregnancy, and self-reporting having a stable partner. Women who were unable to provide consent due to mental illness at baseline, or those whose partner was absent for more than six months a year or whose partner had been tested for HIV during the current pregnancy were not eligible. Enrolled women were randomized to receive either Standard post-test HIV Counseling (SC arm) or the COC intervention (COC arm) [25]. Specifically trained HIV counselors delivered the COC intervention. COC sessions included role-play to develop women’s communication skills, especially to discuss HIV and sexual issues with their partner. Women were also encouraged to invite their partner for HIV testing [29] and couples HIV counseling. All women responded to three face-to-face questionnaires: at baseline, before HIV testing (T0), within two to eight weeks after post-test HIV counseling (T1) and six months post-partum (T2).

### Study population

This analysis is restricted to the three low HIV prevalence countries (The Dominican Republic, Georgia and India) and to women randomized in the COC group. We excluded women who had missing data for study variables.

### Variables of interest

Our outcome of interest was the uptake of couples HIV counseling at the end of the trial, defined as pre-test HIV counseling received together by both partners (even if the couple did not receive couples HIV testing or couples post-test HIV counseling). It was measured from two main sources: i/women self-reports within the questionnaires administered at T1 and T2 and ii/ a trial impact form in which all events of interest for the trial (partner HIV testing and couples HIV counseling in particular) were notified by local trial coordinators. We assumed that women not seen at T1 or T2 and without any information in the trial impact form did not receive couples HIV counseling during the study period.

Individual and couple-related characteristics were included in our analysis. Individual characteristics included age and educational level of the pregnant woman, women’s HIV testing history and reported partner’s HIV testing history. Couple-related characteristics included age and educational difference between the two partners, remunerated activity in the couple, cohabitation, duration of the relationship, report to be accompanied by partner to ANC, perception of partner support for pregnancy, report of emotional, verbal abuse or physical abuse at least once in the last month, and report of past discussion within the couple about HIV and condom use.

### Statistical analysis

We described the uptake of couples HIV counseling at the end of the study for each study site/country. Individual and couple characteristics at baseline were described in terms of numbers and frequencies. Unadjusted (univariable) and multivariable logistic regression models for each study site were used to assess the association between the uptake of couples HIV counseling and the individual and couple-related characteristics. We used a likelihood ratio test (LRT) to screen for variables to be included in the adjusted (multivariable) analyses. Factors that were significant at *P* < 0.25 in unadjusted analyses and woman age as confounder were included in the multivariable model. Final models were retained using a backward elimination approach and after checking for confounding factors. Analyses were carried out using SAS version 9.3.

### Ethics statement

The Prenahtest study was approved by Comité de Ética Indepediente, Fundación Dominicana de Infectología (9 April 2007, Dominican Republic), IRB 00006752 of Maternal and Child Care Union (13 November 2008, Georgia), Independent Ethics Committee for Prayas Health Group (27 March 2007, India). Participants were assigned identification numbers and all questionnaires and process forms were labeled with matching numbers to maintain confidentiality.

## Results

### Description of the study population

**Fig. 1:**
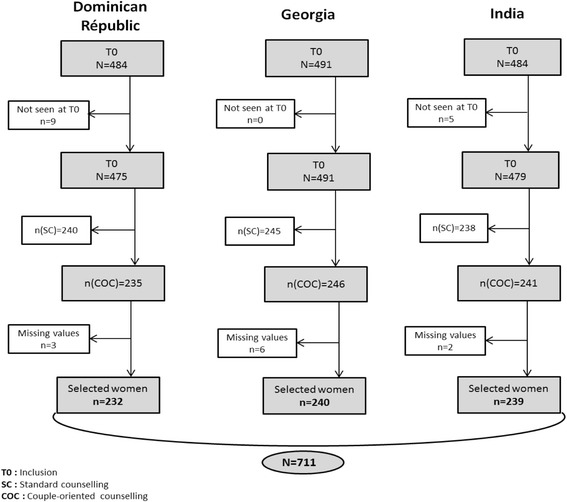
Selection of the study population. Prenahtest ANRS 12127 trial

Overall, 1459 pregnant women were recruited and randomized in the three Prenahtest trial study sites, and 1445 of them responded to the baseline questionnaire. We excluded 723 women randomized to the SC groups and 11 with missing data. A total of 711 pregnant women were included in the analysis, among which 232 in the Dominican Republic, 240 in Georgia and 239 in India (Fig. 1).

Individual and couple characteristics at baseline are described in Table 1.

**Table 1:** Baseline characteristics of women who received couple-oriented post-test HIV counseling. Prenahtest ANRS 12127 trial

	Dominican Republic (*N* = 232)		Georgia (*N* = 240)		India (*N* = 239)	
	*n*	%	*n*	%	*n*	%
Individual characteristics						
Age (year) median (IQR)	21 (19–27)		25 (22–30)		21 (20–23)	
Age (year)						
16–20	75	32.4	22	9.2	56	23.4
20–24	78	33.6	83	34.5	141	59.1
25–29	42	18.1	69	28.8	34	14.2
> =30	37	15.9	66	27.5	8	3.3
Educational level						
None, primary and college	110	47.4	.	.	113	47.2
Secondary	100	43.1	70	29.2	96	40.2
Tertiary	22	9.5	170	70.8	30	12.6
Previously tested for HIV						
Yes	134	57.8	91	37.9	81	33.9
No	98	42.2	149	62.1	158	66.1
Perception of risk of HIV infection from current partner						
Yes	30	12.8	43	17.5	7	2.9
No	168	71.5	157	63.8	218	90.5
Doesn’t Know	37	15.7	46	18.7	16	6.6
Partner ever tested for HIV						
Yes	96	41.4	22	9.2	21	8.8
No	75	32.3	156	65.0	199	83.3
Doesn’t know	61	26.3	62	25.8	19	7.9
Partner alcohol consumption						
Never/Occasionally	226	96.2	229	93.1	225	93.4
Frequently	9	3.8	17	6.9	16	6.6
Couple characteristics						
Marital status						
Single	21	9.0	6	2.5	.	.
Free union	196	84.5	40	16.7	.	.
Married	15	6.5	194	80.8	239	100.0
Educational level difference with partner						
Woman more educated than partner	90	38.8	58	24.2	71	29.6
Partner more educated than woman (<= 2y)	63	27.1	141	58.7	103	43.2
Partner more educated than woman (> 2y)	48	20.7	41	17.1	65	27.2
Doesn’t know	31	13.4	.	.	.	.
Age difference with partner						
Woman older than partner	35	15.1	45	18.8	5	2.1
Partner older than woman (gap <5y)	83	35.8	120	50.0	125	52.3
Partner older than woman (gap >5y)	88	37.9	75	31.2	86	36.0
Doesn’t know	26	11.2	.	.	23	9.6
Remunerated activity within couple						
Woman or both	55	23.7	71	29.6	49	20.5
Partner only	162	69.8	123	51.3	186	77.8
None of them	15	6.5	46	19.1	4	1.7
Cohabitation with partner						
Not cohabiting with partner	30	12.9	15	6.3	18	7.5
Cohabiting with partner only	178	76.7	115	47.9	97	40.6
Cohabiting with partner and family-in-law members	24	10.4	110	45.8	124	51.9
Relationship duration (years)						
< 1	51	22.0	77	32.1	56	23.4
1–5	124	53.4	113	47.1	144	60.3
> 5	57	24.6	50	20.8	39	16.3
Woman accompanied by partner to antenatal care						
Yes	64	27.6	91	37.9	105	43.9
No	168	72.4	149	62.1	134	56.1
Planning of the current pregnancy with partner						
Planned	98	42.2	174	72.5	195	81.6
Not planned	134	57.8	66	27.5	44	18.4
Perception of partner support during the pregnancy						
Very supportive	146	62.9	92	38.3	164	68.6
Supportive/not supportive enough	86	37.1	148	61.7	75	31.4
Emotional or verbal abuse from partner at least once in the last month						
Yes	112	48.3	62	25.8	47	19.7
No	120	51.7	178	74.2	192	80.3
Physical abuse from partner at least once in the last month						
Yes	24	10.3	4	1.7	39	16.3
No	208	89.7	236	98.3	200	83.7
Ever discussed about HIV with partner						
Never	89	38.4	140	58.4	129	54.1
On general terms	53	22.8	92	38.3	83	34.7
On personal terms	90	38.8	8	3.3	27	11.2
Condom use with partner and discussion about it						
Already used and discussed	100	43.1	72	30.0	64	26.8
Already used but never discussed	33	14.2	6	2.5	5	2.1
Never used but already discussed	38	16.4	37	15.4	60	25.1
Never used and never discussed	61	26.3	125	52.1	110	46.0

Dominican and Indian women were aged 21 years in median (interquartile range (IQR) = [19–27] and IQR = [20–23], respectively) and most of them had completed less than 13 years of education (91.5% and 87.5%, respectively). Georgian women were aged 25 years in median (IQR = [22–30]) and most of them (70.8%) had completed at least 13 years of education.

In Georgia, 80% of women reported being in a married relationship. All women enrolled in India were married. In the Dominican Republic, >84% reported living in free union (i.e. living with a partner without legal or religious recognition). Women often lived with both their partner and his family members in Georgia (45%) and India (52%), while in Dominican Republic women were more likely to live with their partner only (i.e. as a nuclear family) (76.7%).

In all three sites, less than one third of pregnant women were employed at the time of enrolment and up to 20% of both partners were not working in Georgia. Few women had a higher educational level than their partner (respectively 24%, 29% and 39% of women in Georgia, India and the Dominican Republic).

In all three countries, women had occasionally been accompanied by their partner to their first ANC visit: 44% in India, 38% in Georgia and 28% in the Dominican Republic. Approximately two thirds of women in the Dominican Republic and India perceived their partner as very supportive of their pregnancy. 48.3% of women in the Dominican Republic reported experiencing emotional or verbal abuse from their partner at least once in the last month prior enrolment. This proportion was lower in Georgia (25.8%) and India (19.7%). Physical abuse by the partner in the last month was reported by 16.3% of women in India, 10.3% in the Dominican Republic and 1.7% in Georgia. Frequent alcohol consumption by their partner was reported by 3.8% of women in the Dominican Republic, 6.9% in Georgia and 6.6% in India.

Previous HIV testing was reported by 37.9% of women in Georgia, 33.9% in India, and 27.8% in the Dominican Republic. HIV testing history among men, as reported by women, was below 10% in Georgia and India, and 41.4% in Dominican Republic.

At enrolment, 58.3% of women in Georgia, 54.1% in India and 38.4% in the Dominican Republic reported never having discussed about HIV with their partner; 43.1% of women in the Dominican Republic, 30.0% in Georgia and 26.8% in India reported ever having used condoms for HIV prevention with their partner.

### Uptake of couples HIV counseling at the end of the trial

**Fig. 2:**
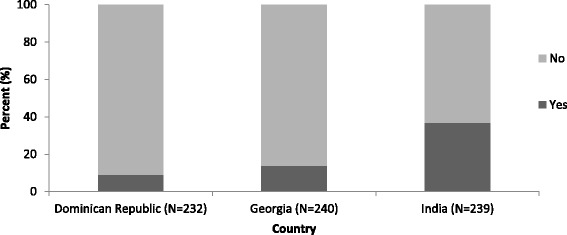
Uptake of couples HIV counseling among women who received couple-oriented post-test counseling. Prenahtest ANRS 12127 trial

Among the 711 women included in this analysis, 131 were seen at T1 only, 507 at T1 and T2, 11 at only T2 and 62 were not seen neither at T1 nor T2. A total of 141 women (19.8% of all included women) reported receiving couples HIV counseling during the trial, either at T1 (114 women), or at T2 (28 women). The uptake of couples HIV counseling was uneven by country: 9.1% (21 couples) in the Dominican Republic, 13.8% (33 couples) in Georgia and 36.8% (88 couples) in India (Fig. 2).

### Individual and couples characteristics associated with the uptake of couples HIV counseling.

Unadjusted (univariable) analyses are presented in Table 2 and adjusted (multivariable) analyses in Table 3.

**Table 2:** Univariable analysis of factors associated with the uptake of couples HIV counseling. Prenahtest ANRS 12127 trial

	Dominican Republic (*N* = 232)			Georgia (*N* = 240)			India (*N* = 239)		
	*n* (%)	OR (95% CI)	*p*-value	*n* (%)	OR (95% CI)	*p*-value	*n* (%)	OR (95% CI)	*p*-value
Individual characteristics									
Age (year)			0.332			0.935			0.084
20–24	4(5.1)	1.00		12(14.5)	1.00		54(38.3)	1.00	
16–20	9(12.0)	2.52 [0.78–9.66]		3(13.6)	0.93 [0.20–3.31]		22(39.3)	1.04 [0.55–1.96]	
25–29	3(7.1)	1.42 [0.27–6.77]		8(11.6)	0.77 [0.29–2.00]		7(20.6)	0.49 [0.16–0.98]	
> =30	5(13.5)	2.89 [0.72–12.35]		10(15.2)	1.06 [0.42–2.63]		5(62.5)	2.68 [0.63–13.51]	
Educational level			0.999			0.026			0.890
Secondary	9(9.0)	1.00		4(5.7)	1.00		37(38.5)		
None, primary and college	10(9.1)	1.01 [0.39–2.65]		–	–		40(35.4)	0.87 [0.50–1.54]	
Tertiary	2(9.1)	1.01 [0.15–4.31]		29(17.1)	3.39 [1.27–11.79]		11(36.7)	0.92 [0.39–2.13]	
Previously tested for HIV			0.685			0.338			0.422
Yes	13(9.7)	1.00		15(16.5)	1.00		27(33.3)	1.00	
No	8(8.2)	0.82 [0.32–2.05]		18(12.1)	0.69 [0.33–1.48]		61(38.6)	1.26 [0.72–2.23]	
Perception of risk of HIV infection from her current partner			0.884			0.115			0.478
No	15(8.9)	1.00		21(13.4)	1.00		84(38.5)	1.00	
Yes	4(13.3)	1.57 [0.42–4.73]		5(11.6)	0.85 [0.27–2.25]		2(28.6)	0.64 [0.09–3.03]	
Doesn’t Know	2(5.4)	0.58 [0.09–2.19]		7(15.2)	1.16 [0.43–2.82]		4(25.0)	0.53 [0.14–1.58]	
Partner ever tested for HIV			0.381			0.035			0.704
Yes	10(10.4)	1.00		7(31.8)	1.00		6(28.6)	1.00	
No	8(10.7)	1.03 [0.37–2.74]		16(10.3)	0.24 [0.09–0.72]		75(37.7)	1.51 [0.59–4.39]	
Doesn’t Know	3(4.9)	0.44 [0.10–1.53]		10(16.1)	0.41 [0.13–1.30]		7(36.8)	1.46 [0.39–5.68]	
Partner alcohol consumption			0.8213			NC			0.0948
Never/Occasionally	20(8.8)	1.00		33(14.1)	1.00		87(38.7)	1.00	
Frequently	1(11.1)	1.29 [0.07–7.55]		0(0.0)	NC		3(18.7)	0.37 [0.08–1.17]	
Couple characteristics									
Educational level difference with partner			0.654			0.004			0.463
Partner more educated than woman (<= 2 y)	8(12.7)	1.00		14(9.9)	1.00		34(33.0)	1.00	
Woman more educated than partner	7(7.8)	0.58 [0.19–1.70]		16(27.6)	3.45 [1.56–7.77]		30(42.3)	1.48 [0.79–2.78]	
Partner more educated than women (> 2 y)	3(6.3)	0.46 [0.10–1.69]		3(7.3)	0.716[0.16–2.34]		24(36.9)	1.19 [0.62–2.27]	
Doesn’t know	3(9.7)	0.74 [0.15–2.77]							
Age difference with partner			0.603			0.193			0.098
Partner older than woman (gap <5 y)	5(6.0)	1.00		13(10.8)	1.00		44(35.2)	1.00	
Woman older than partner	4(11.4)	2.01 [0.47–8.09]		10(22.2)	2.35 [0.93–5.83]		2(40.0)	1.23 [0.16–7.67]	
Partner older than woman (gap >5 y)	10(11.4)	2.00 [0.68–6.67]		10(13.3)	1.26 [0.51–3.04]		28(32.6)	0.89 [0.49–1.58]	
Unknown	2(7.7)	1.30 [0.18–6.47]					14(60.9)	2.86 [1.16–7.38]	
Remunerated activity within the couple			0.767			0.044			0.391
Partner only	15(9.3)	1.00		13(10.6)	1.00		65(34.9)	1.00	
Woman or both	4(7.3)	0.77 [0.21–2.23]		16(22.5)	2.46 [1.11–5.56]		22(44.9)	1.52 [0.80–2.87]	
None of them	2(13.3)	1.51 [0.22–6.18]		4(8.7)	0.80 [0.22–2.43]		1(25.0)	0.62 [0.03–4.95]	
Cohabitation			0.518			0.471			0.067
With partner only	18(10.1)	1.00		19(16.5)	1.00		35(36.1)	1.00	
Without partner	2(6.7)	0.63 [0.10–2.37]		2(13.3)	0.78 [0.12–3.13]		2(11.1)	0.22 [0.03–0.84]	
With partner and family-in-law members	1(4.2)	0.39 [0.02–2.02]		12(10.9)	0.612 [0.28–1.33]		51(41.1)	1.24 [0.72–2.15]	
Relationship duration (year)			0.069			0.821			0.467
1–5	9(7.4)	1.00		14(12.4)	1.00		52(36.1)	1.00	
< 1	9(17.6)	2.74 [1.01–7.47]		12(15.6)	1.30 [0.56–3.00]		24(42.9)	1.33 [0.70–2.49]	
> 5	3(5.4)	0.71 [0.15–2.49]		7(14.0)	1.15 [0.41–2.98]		12(30.8)	0.78 [0.36–1.65]	
Accompanied by partner to antenatal care			0.011			0.843			<0.001
Yes	11 (17.7)	1.00		12(13.2)	1.00		55(52.4)	1.00	
No	10(6.1)	0.30 [0.12–0.76]		21(14.1)	1.08 [0.51–2.38]		33(24.6)	0.30 [0.17–0.51]	
Planning of the current pregnancy			0.952			0.975			0.262
Planned	9(9.4)	1.00		24(13.8)	1.00		75(38.5)	1.00	
Not planned	12 (9.1)	0.97 [0.39–2.48]		9(13.6)	0.99 [0.41–2.19]		13(29.5)	0.67 [0.32–1.34]	
Perception of partner support during the pregnancy			0.034			0.366			0.106
Very supportive	18(12.3)	1.00		15(16.3)	1.00		66(40.2)	1.00	
Supportive/ not enough	3(3.5)	0.26 [0.73–0.90]		18(12.2)	0.71 [0.34–1.49]		22(29.3)	0.62 [0.34–1.11]	
Emotional or verbal abuse from partner at least once in the last month			0.054			0.300			0.433
Yes	6(5.4)	1.00		11(17.7)	1.00		15(31.9)	1.00	
No	15(12.5)	2.52 [0.98–7.30]		22(12.4)	0.65 [0.30–1.48]		73(38.0)	1.31 [0.67–2.64]	
Physical abuse from partner at least once in the last month			0.334			–			0.342
Yes	1(4.2)	1.00		–	–		17(43.6)	1.00	
No	20(9.6)	2.44 [0.47–44.90]		–	–		71(35.5)	0.71 [0.36–1.44]	
Ever discussed about HIV in the couple			0.410			0.004			0.788
On general terms	4(7.5)	1.00		19(20.7)	1.00		29(34.9)	1.00	
Never	6(6.7)	0.88 [0.24–3.61]		11(7.9)	0.33 [0.14–0.72]		50(38.8)	1.18 [0.67–2.10]	
On personal terms	11(12.2)	1.70 [0.55–6.42]		3(37.5)	2.30 [0.44–10.27]		9(33.3)	0.93 [0.36–2.29]	
Condom use with partner and discussion about it			0.134			0.956			0.523
Already used and discussed	5(5.0)	1.00		10(13.9)	1.00		19(29.7)	1.00	
Already used but never discussed	2(6.1)	1.22 [0.17–6.01]		1(16.7)	1.24 [0.06–8.82]		2(40.0)	1.58 [0.20–10.27]	
Never used but already discussed	6(15.8)	3.56 [1.01–13.13]		6(16.2)	1.20 [0.38–3.54]		22(36.7)	1.37 [0.65–2.92]	
Never used and never discussed	8(13.1)	2.87 [0.91–9.91]		16(12.8)	0.91 [0.39–2.19]		45(40.9)	1.64 [0.86–3.21]	

*OR* Crude Odds-Ratio, *95% CI* 95% Confidence Interval, *ANC* Antenatal Care visit, *NC* Not Calculable

**Table 3:** Multivariable analysis of factors associated with the uptake of couples HIV counseling. Prenahtest ANRS 12127 trial

	Dominican Republic (N = 232)			Georgia (N = 240)			India (N = 239)		
	*n* (%)	AOR (95% CII)	*p*-value	*n* (%)	AOR (95%)	*p*-value	*n* (%)	AOR (95% CI)	*p*-value
Educational level difference with partner						0.004			
Partner more educated than women (<=2 y)				14 (9.9)	1.00				
Women more educated than partner				16 (27.6)	3.56 [1.53–8.26]				
Partner more educated than women (> 2 y)				3 (7.3)	0.72 [0.15–2.33]				
Accompanied by partner to ANC			0.008						<0.001
No	10 (6.1)	1.00					33 (24.6)	1.00	
Yes	11 (17.7)	3.85[1.40–10.56]					55 (52.4)	3.57 [2.03–6.28]	
Perception of partner support during the pregnancy			0.049						
Very supportive	18(12.3)	1.00							
supportive/ not enough	3(3.5)	0.26 [0.07–0.98]							
Emotional or verbal abuse from partner at least once in the last month			0.090						
Yes	1(4.2)	1.00							
No	20(9.6)	2.95 [0.95–9.17]							
Ever discussed about HIV in the couple						0.006			
On general terms				19 (20.7)	1.00				
Never				11 (7.9)	0.32 [0.13–0.74]				
On personal terms				3 (37.5)	2.85 [0.56–14.46]				
Condom use with partner and discussion about it			0.046						
Already used and discussed	5(5.00)	1.00							
Already used but never discussed	2 (6.10)	1.55[0.26–8.68]							
Never used but already discussed	6 (15.8)	5.37[1.59–21.80]							
Never used and never discussed	8 (13.1)	3.44[1.06–11.97]							

*AOR* Adjusted Odds-Ratio, *95% CI* 95% Confidence Interval, *ANC* Antenatal Care visit, *n (%)* number of couple counseling events (proportion of couple counseling per modality). Models adjusted on Women age

In Dominican Republic, the multivariable analysis showed that the uptake of couples HIV counseling was significantly more likely among women who were accompanied by their partner to their first ANC visit (17.7% versus 6.1%, adjusted Odds-Ratio (aOR) = 4.10, 95% Confidence Interval (95% CI) = [1.51, 11.13]). Couples HIV counseling was also more likely among women who had never used a condom with their partner but had ever discussed it (15.8%) and among those who had never used a condom and never discussed it with the partner (13.1%), compared to those who declared ever having used a condom and ever having discussed it with their partner (5.0%) (respectively, aOR = 6.17, 95% CI = [1.57, 24.20] and aOR = 3.67, 95% CI = [1.07, 12.63]). The uptake of couples HIV counseling was less likely among women who perceived their partner as normally or less supportive of the current pregnancy compared to those who perceived their partner as supportive (3.5% versus 12.3%, aOR = 0.26, 95% CI = [0.73–0.90]). (Table 2).

In Georgia, the multivariable analysis showed that the uptake of couples HIV counseling was significantly more likely among women with a higher educational level than their partner, compared to the contrary (27.6% versus 9.9%, aOR = 3.45, 95% CI = [1.56, 77]). The uptake of couples HIV counseling was less likely among women who had never discussed about HIV with their partner, compared to those who had had a discussion about HIV only on general terms (7.9% versus 20.7%, aOR = 0.33, 95% CI = [0.14, 0.72]) (Table 2).

In India, in the multivariable analysis, the uptake of couples HIV counseling was more likely among women who had been accompanied by their partner to their first ANC visit (52.4% versus 24.6%, OR = 3.37, 95% CI = [1.95, 5.83]) (Table 2).

## Discussion

Our results showed that the uptake of couples HIV counseling within the Prenahtest trial, among women who specifically received an intervention dedicated to supporting a couples approach to HIV counseling and testing, was low in the Dominican Republic and Georgia (respectively 9.1% and 13.8%) and a little higher in India (36.8%). Further, the uptake of couples HIV counseling was overall lower than what we reported for partner HIV testing [29], highlighting the challenges of providing couples HIV services in antenatal settings.

Our analysis also provided insight into the profile of women and their partner who engaged in couples HIV counseling, which varied substantially across study sites. In Georgia, couples in which the woman was more educated than her partner were more likely to receive couples HIV counseling. The role of education level on the acceptability and impact of behavioral interventions has been shown in several low HIV prevalence contexts, including in India [30, 31]. More educated woman may have had stronger confidence to initiate discussion about HIV and better negotiation skills within their couple relationship to encourage their partner to receive couples HIV counseling. Previous discussions about HIV within the couple also contributed to a better uptake of couples HIV counseling. Thus, promoting communication within couples and bringing information about HIV to both couple members might contribute to improve couples HIV counseling uptake in the Georgian context.

In India, only one characteristic was associated with the uptake of couples HIV counseling: the fact that the woman was accompanied by the partner to ANC. The presence of men in ANC seems to have triggered some form of “provider-initiated couples HIV counseling”, which proved highly effective. These findings are comparable with those recently reported in a pilot project in 15 hospitals in Thailand, with a very high uptake of couples HIV counseling among women accompanied by their partner to ANC [32].

In the Dominican Republic, in addition to women being accompanied to ANC by their partner, women’s perception of a strong involvement of their partner in the pregnancy was associated with the uptake of couples HIV counseling. This association was not found in the two others countries or in the literature. Also, couples that had never used a condom were more likely to receive couples HIV counseling than those who had already used condoms and discussed about it. These results may be explained by the fact that couples with little information on HIV might feel less anxious about HIV screening. This hypothesis is supported by the results of a study conducted in Kenya in 2001 that found that men who opted for couples HIV counseling had less knowledge about HIV than those who chose individual HIV counseling [33].

One of the limits of our study may be that we restricted our analysis to women from the intervention group. This choice was made based on methodological reasons, as in Georgia, no women from the SC group had reported receiving couples HIV counseling. The fact that we excluded one of the four study sites may have led to a partial appreciation of Prenahtest results, but this choice was motivated by epidemiological reasons, as Cameroon has a much higher HIV prevalence than the three other sites, with prevalence rates below 1%. Finally, the fact that all data were self-reported by women may have led in part to an information bias regarding partner and couple characteristics.

Overall however, this multi-site analysis allowed investigating couples HIV counseling in three very different socio-demographic contexts, while ensuring a standardized approach in comparing results between sites. Our analysis added to the scarce literature on couples HIV prevention in low-income, low HIV prevalence and non-African cultural settings.

These findings suggest overall that improving the uptake of couples HIV counseling will require substantial structural changes, such as improving women’s education level or encouraging an evolution in gender norms to facilitate the presence of men in health services for sexual and reproductive health and to strengthen the ability of women to discuss HIV with their partners. The urgent need for an improved HIV response does not fit well with such long-term behavior change. However, the current changes in the HIV care landscape with the generalization of ART, which will impact on individual health, may sooner impact couple relationships and contribute to higher acceptability and uptake of biomedical and behavioral interventions that address couple concerns.

## Conclusion

Although the efficacy of the COC intervention tested within the Prenahtest trial was limited in terms of uptake of couples HIV counseling, we showed previously its value in improving partner HIV testing [29] and short-term communication about HIV within couples [34]. Other couples-centered strategies have been evaluated to increase the uptake of HIV testing and the adoption of HIV prevention strategies within couples. These are essentially community-based interventions such as home-based visits [35–37], and clinic-based interventions such as invitation letters from health care workers to partners [38]. The efficacy of women-delivered HIV self-test in reaching men, who remain underserved by existing services, is about to be evaluated [39]. Combined strategies, tailored to the socio-cultural context, will be critical to improve the uptake of couples HIV counselling and testing and overall interventions for HIV prevention among couples in low to medium HIV prevalence countries.

## Abbreviations

ANC: Antenatal care
ANRS: Agence Nationale de Recherches sur le SIDA et les hépatites virales
AOR: Adjusted odds-ratio
ART: Antiretroviral treatment
CI: Confidence interval
COC: Couple-Oriented post-test HIV Counselling
OR: Crude Odds-Ratio
PMTCT: Prevention of mother-to-child transmission
PreP: Pre-exposure Prophylaxis
SC: Standard post-test HIV Counselling
